# Locoregional Flaps for the Reconstruction of Midface Skin Defects: A Collection of Key Surgical Techniques

**DOI:** 10.3390/jcm12113700

**Published:** 2023-05-26

**Authors:** Giovanni Salzano, Francesco Maffìa, Luigi Angelo Vaira, Umberto Committeri, Chiara Copelli, Fabio Maglitto, Alfonso Manfuso, Vincenzo Abbate, Paola Bonavolontà, Alfonso Scarpa, Luigi Califano, Giovanni Dell’Aversana Orabona

**Affiliations:** 1 Maxillofacial Surgery Operative Unit, Department of Neurosciences, Reproductive and Odontostomatological Sciences, Federico II University of Naples, 80131 Naples, Italy; francesco.maffia@gmail.com (F.M.); umbertocommitteri@gmail.com (U.C.); vincenzo.abbate@unina.it (V.A.); paola.bonavolonta@unina.it (P.B.); califano@unina.it (L.C.); giovanni.dellaversanaorabona@unina.it (G.D.O.); 2 Maxillofacial Surgery Operative Unit, Department of Medicine, Surgery and Pharmacy, University of Sassari, 07100 Sassari, Italy; lavaira@uniss.it; 3 Biomedical Science Department, PhD School of Biomedical Science, University of Sassari, 07100 Sassari, Italy; 4 Maxillofacial Surgery Operative Unit, Department of Interdisciplinary Medicine, Aldo Moro University of Bari, 70120 Bari, Italy; chiara.copelli@uniba.it (C.C.); fabio.maglitto@policlinico.ba.it (F.M.); alfonso.manfuso@policlinico.ba.it (A.M.); 5 Department of Medicine and Surgery, University of Salerno, 84081 Salerno, Italy; alfonsoscarpa@yahoo.it

**Keywords:** head and neck reconstruction, midface skin defects, midface locoregional flaps, pedunculated flaps

## Abstract

Background: The reconstruction of midface skin defects represents a challenge for the head and neck surgeon due to the midface’s significant role in defining important facial traits. Due to the high complexity of the midface region, there is no possibility to use one definitive flap for all purposes. For moderate defects, the most common reconstructive techniques are represented by regional flaps. These flaps can be defined as donor tissue with a pedunculated axial blood supply not necessarily adjacent to the defect. The aim of this study is to highlight the more common surgical techniques adopted for midface reconstruction, providing a focus on each technique with its description and indications. Methods: A literature review was conducted using PubMed, an international database. The target of the research was to collect at least 10 different surgical techniques. Results: Twelve different techniques were selected and cataloged. The flaps included were the bilobed flap, rhomboid flap, facial-artery-based flaps (nasolabial flap, island composite nasal flap, retroangular flap), cervicofacial flap, paramedian forehead flap, frontal hairline island flap, keystone flap, Karapandzic flap, Abbè flap, and Mustardè flap. Conclusions: The study of the facial subunits, the location and size of the defect, the choice of the appropriate flap, and respect for the vascular pedicles are the key elements for optimal outcomes.

## 1. Introduction

The reconstruction of midface skin defects represents a challenge for the head and neck surgeon due to its renowned role in defining important facial traits [1]. Midfacial skin defects can originate from neoplasm asportation or be secondary to facial trauma [2]. The term “midface” refers to several anatomical sub-regions including the infraorbital region, malar region, and nasolabial region [3]. The division of the face into facial aesthetic subunits represents a valid method to delimitate the face in regions with similar characteristics [4]. A particularity of these areas is represented by how they show different thicknesses and layers, even among contiguous regions, complicating reconstruction planning [5,6]. Indeed, the functionality and high aesthetical consideration of the midface require the surgeon to find innovative solutions to restore form and function [7]. In the surgical treatment of midface skin defects, a meticulous preoperative assessment is required to properly assess the anticipated gap and to determine the proper reconstructive plan [3]. Split-thickness skin grafts, full-thickness skin grafts, pedunculated flaps, and free flaps are the main surgical options in soft tissue defect reconstruction [8]. Due to the high complexity of the midface region, there is no possibility to use one definitive flap for all purposes, so expanding our surgical arsenal is necessary to overcome reconstruction difficulties [2]. For moderate defects, the most common reconstructive techniques are represented by regional flaps. These flaps can be defined as donor tissue with a pedunculated axial blood supply not necessarily adjacent to the defect [1]. To date, there is no consensus on the most appropriate flap design for specific defects, especially in defects located across more midfacial subunits [9]. The most common pedicle in many of the flaps for midface reconstruction is represented by the facial artery: thanks to its course and its collateral branches, it allows almost complete coverage of the whole region [10]. The choice of locoregional flaps is also supported by favorable skin color- and texture-match characteristics [11]. The continuous development of surgical techniques has led to a wide armamentarium of different midfacial regional flaps, related to high facial vascularization [12]. The aim of this study is to highlight the more common surgical techniques adopted for the reconstruction of a complex area such as the midface. The authors provide a focus on each technique with its description and indications based on defect size.

## 2. Materials and Methods

### 2.1. Literature Review

Initial background research was conducted to outline the conceptual foundations of the study: the reconstruction of midface soft tissue. A literature review was conducted using PubMed, an international database. The keywords used were: “head and neck surgery”, “head and neck reconstruction”, “head and neck skin defects”, and “midface skin defects”. The database used was screened using the keywords in combination with “surgical flaps”, and “locoregional flaps” included in the research string.

### 2.2. Study Selection

The review of the literature was conducted in November 2022. The researchers decided to not include a chronological limit to the research. The target of the research was to collect at least 10 different surgical techniques with diverse complexity indexes. Adopted inclusion criteria were (a) articles written in English; (b) technique studies, retrospective studies, and prospective studies; (c) studies describing the surgical reconstruction of a midface skin defect; and (d) the adoption of pedunculated flaps harvested with only soft tissue. Exclusion criteria were (a) articles not describing the surgical technique adopted; (b) articles involving more complex or multilayered defects; and (c) the adoption of free flaps. The studies matching the inclusion criteria were recognized and were read in full to perform the data extraction.

### 2.3. Surgical Techniques Data Extraction

After individuating the different techniques, data were extracted accounting for complete technique explanations, descriptions of application areas, and descriptions of the defect’s diameter. When the same technique was reported in more than one study, the most precise was preferred and selected. All data were stored in a digital database including the technique name, the type of the adopted flap, the main midface region treated, suggested secondary regions, and skin defect size (small, medium, large). The classification in sizes followed a centimetric division found in the literature: 1.5–2 cm for “small”, 2–3 cm for “medium”, and >3 cm for “large”. Additional or missing information was compared among similar studies and collected in the digital database.

## 3. Results

Among the studies obtained, 12 different techniques were selected and cataloged. The main characteristics of each technique are reported in Table 1.

The type of flap was divided into transpositional (5/12, 41%), rotational (4/12, 33%), and advancement flaps (2/12 16%). Just one flap was considered both rotational and advancement. The cheek and nose areas were the most described regions (both 5/12, 41%), followed by the upper lip area (2/12, 16%). Defect size indications were mostly “medium” (9/12, 75%).

### Surgical Techniques Collection

**Figure 1:**
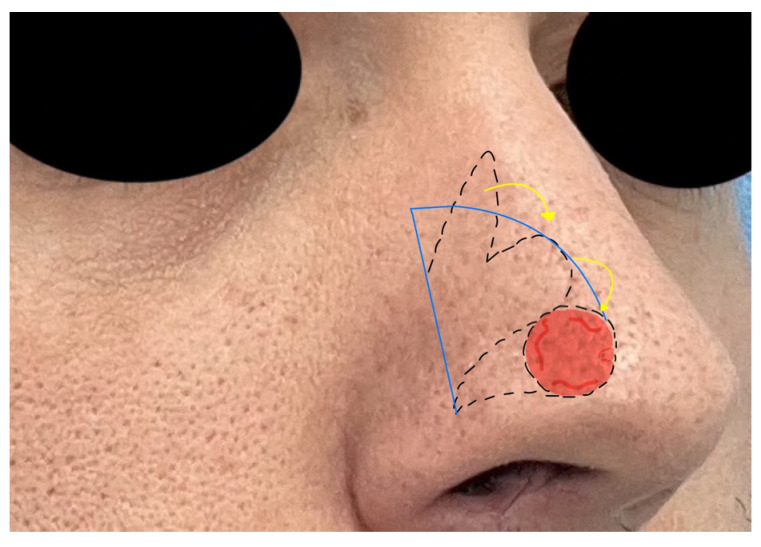
The bilobed flap with its geometrical drawing. Red zone indicate the skin defect, dotted lines indicate cuts, blue lines are geometric landmarks, and yellow arrows indicate flap shifts.

**Figure 2:**
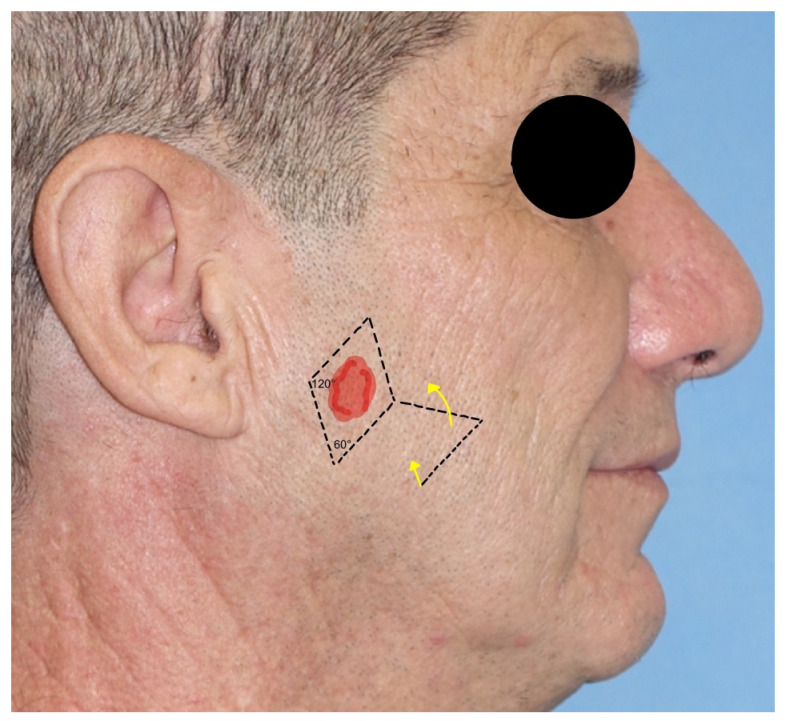
Rhomboid flap with angles and incision lines.

**Figure 3:**
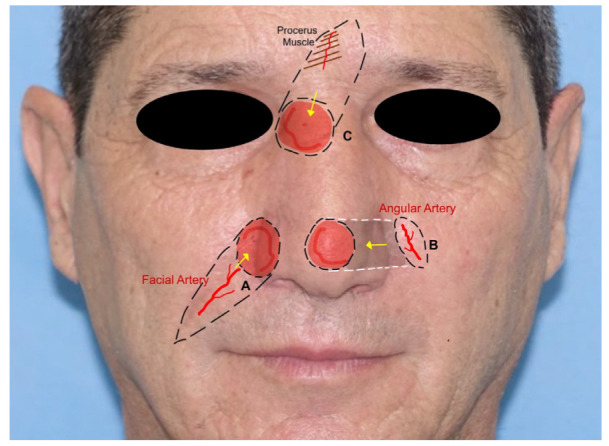
Facial-artery-based flap: (**A**), nasolabial flap, with evidence of the facial artery as the pedicle; (**B**), retroangular flap, with evidence of angular artery as the pedicle, the grey shade indicates the subcutaneous tunneling; (**C**), island composite nasal flap, with exposure of the procerus muscle.

The highlights of the techniques included are described in the following list:

1. *Bilobed flap*: the bilobed flap (BF) is a single pedicle random pattern cutaneous transposition flap composed of two lobes. Its applications are various, including the hand, scalp, foot, and eyelids, but its main adoption is in nasal reconstruction for its excellent color and texture match with adjacent skin. The BF is used for small nasal defects (1.5 cm). Nasal ala defects are approached with a medially based flap, while tip defects are approached with a laterally based one. The surgical technique requires precise measurements: the first measure is the diameter of the defect (D), then the pivot point is placed at least 0.5 D from the defect; from the pivot point, the outer flap edge is obtained by rotating a suture around the pivot point; an inner arch is marked 0.5 D inside the outer arch; and the first lobe is demarcated with a diameter equal to D, while the second lobe is marked with a width 0.5 higher than D (Figure 1). The dissection is performed above the periosteum and perichondrium. The first lobe covers the excision gap and the second lobe will replace the first lobe, while the gap of the second lobe is closed by direct suture [11].

2. *Rhomboid flap*: the rhomboid flap (RF), also known as the Limberg flap, is a transposing rhomboid-shaped flap, used typically for medium-sized defects of the cheek (2–2.5 cm). It can be designed in every direction. The main advantage is that, being adjacent to the defect, it matches the skin color and texture. Its plan is geometrical: the defect is surrounded by a rhomboid with 60° and 120° angles; then, the flap is designed on the short axis (120°), as shown in Figure 2. The Dufourmentel flap is a modification of the Limberg flap and uses angles from 60° to 90° [11,13,14].

3. Facial-artery-based flaps: The *nasolabial flap (NLF*) is the most adopted advancement flap in the reconstruction of the nasal side wall and ala defects and also cheek and upper lip regions. The NLF is used to cover medium defects close to the flap. It is based on the branches of the facial artery, most commonly on the angular artery. It is triangular-shaped, with the base of the triangle direct to the defect, while the apex is directed toward the pedicle. Once the flap is mobilized, the surgical wound is closed in a V-Y fashion [2,15] (Figure 3A).

The *retroangular flap* (RAF) is a transpositional flap used in the reconstruction of the lower half of the nose, the lower eyelid, and the medial cheek. It is based on the angular artery, detected preoperatively by a Doppler. The flap is harvested from the nasolabial fold, with the first incision made in the distal part, to identify the angular pedicle. After isolating the vascular pedicle, the flap is islanded distal to medial with a subfascial dissection. After subcutaneous tunneling, the flap is inserted into the gap [10] (Figure 3B).

The *island composite nasal flap* (ic-NF) is adopted to increase the mobility of the distance covered by the classic nasolabial flap and is harvested using the procerus muscle near the defect as a pedicle. It is commonly used in the upper nasal region, the lower eyelid, and the medial canthal area. The flap is elevated under the muscle on the non-pedicle side and then advanced to the defect area. The donor site is closed with a V-Y technique. It can also be tunneled to reach more distant regions [10,16] (Figure 3C).

**Figure 4:**
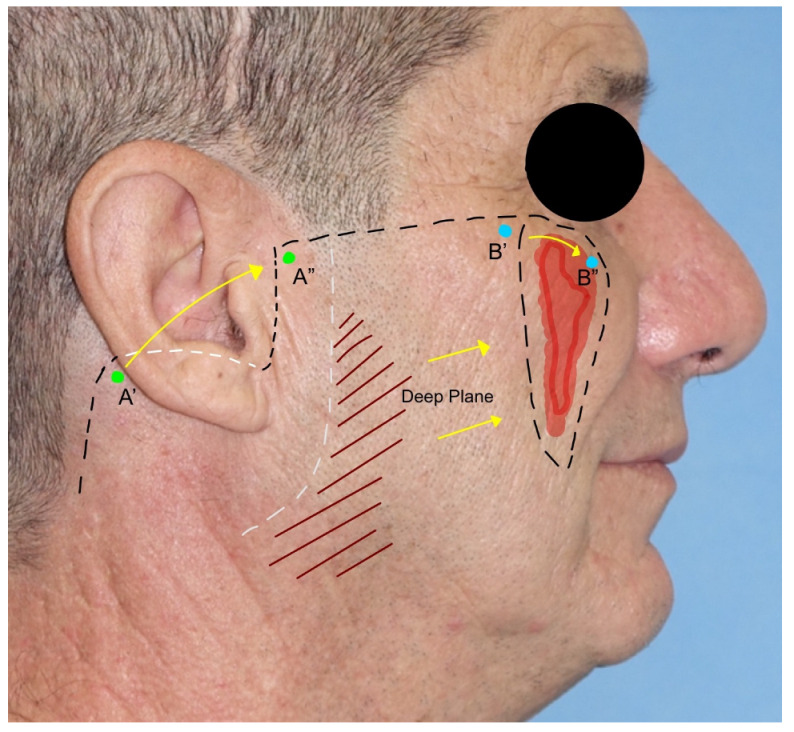
Cervicofacial flap. Part of the incision is hidden behind the ear lobe. During the dissection, a deep-plane incision is needed to permit the advancement of the flap. The skin has been marked with points A and B to show the incision’s advancement.

**Figure 5:**
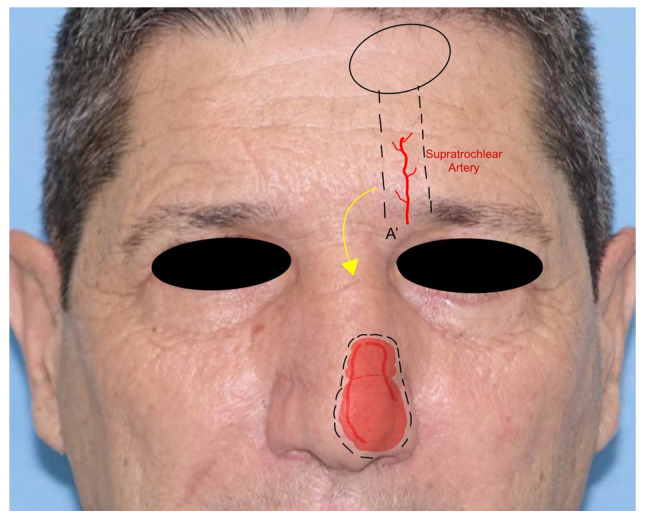
Paramedian forehead flap. The A’ point represents the pivotal point for the flap rotation. The supratrochlear artery is shown as the pedicle of the flap.

**Figure 6:**
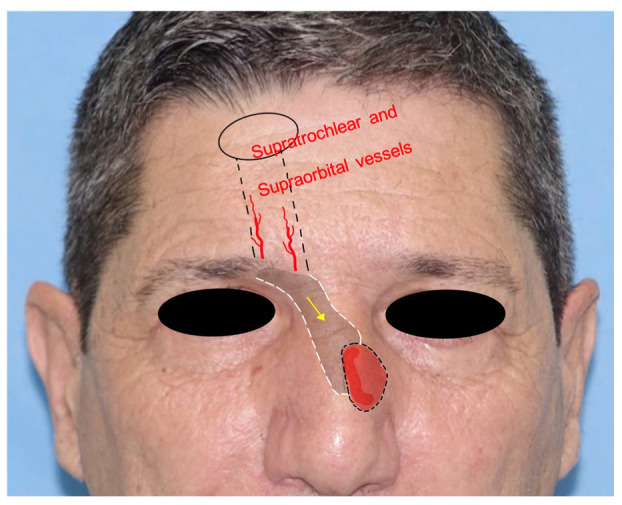
The frontal hairline island flap, with evidence of the vascular pedicle; the grey shade indicates the subcutaneous tunneling.

**Figure 7:**
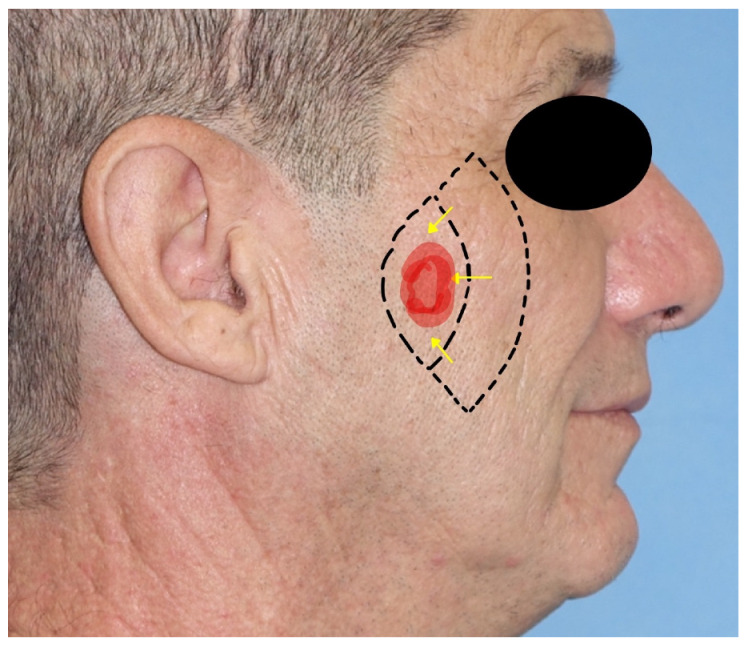
The keystone flap.

**Figure 8:**
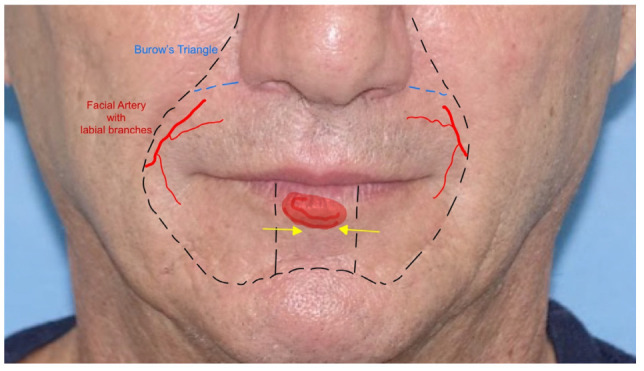
The Karapandzic flap: two Burow triangles are shown. The labial artery pedicle is indicated.

**Figure 9:**
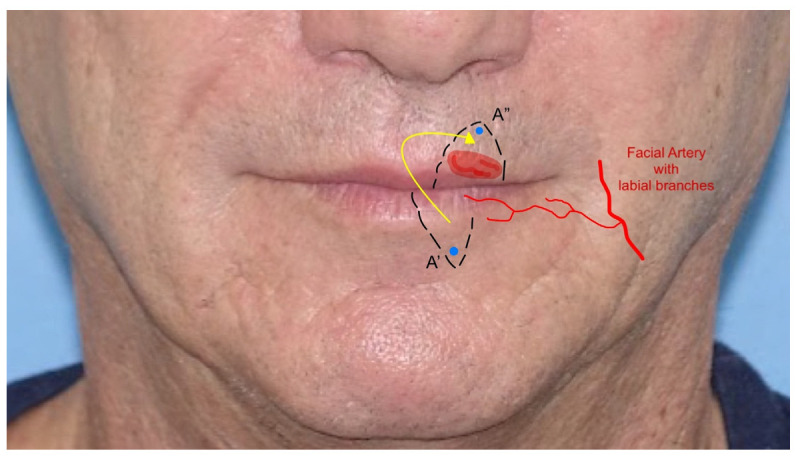
The Abbè flap: point A’ is rotated and inserted in the upper lip, becoming point A”. The branches of the labial artery are indicated.

**Figure 10:**
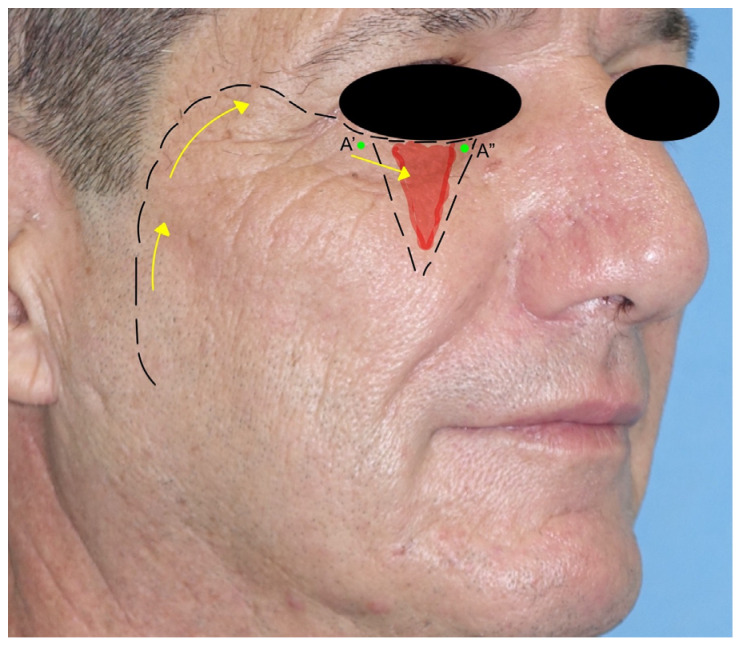
The Mustardè flap: the A’ point is rotated to fill the skin gap till the A” point is reached.

4. *Cervicofacial flap*: the cervicofacial flap is suited for moderate and large (>3 cm) defects of the cheek and is extendible to the periorbital and neck regions. It represents a combination of an advancement and rotational flap that can be moved anteriorly or posteriorly and anteriorly. The incision is a variant of the rhytidectomy approach but continues through the zygomatic arch, the lateral cantus, and the lower eyelid line until the nasolabial groove is reached in its most inferior part. The incision can be prolonged posteriorly, as in a parotidectomy with a horizontal limb. The flap is elevated superficially to the superficial musculoaponeurotic system (SMAS) and the masseteric fascia to avoid facial nerve branch injuries. After rotation and advancement, the donor site is closed by direct suture. Due to the great dimension of the flap, anchoring the flap to the orbital–zygomatic periosteum is necessary [11,17] (Figure 4).

5. *Paramedian forehead flap*: the paramedian forehead flap (PMFF) is an interpolated rotational flap harvested from the forehead and used for reconstructing large and complex defects of the nose. It is harvested based on the blood supply of the supratrochlear artery. The PMFF requires an accurate template of the defect to fill due to the complex three-dimensionality of the nasal region (Figure 4). After the identification of the supratrochlear pedicle by Doppler, the flap is harvested through the skin and subcutaneous muscle in a subfascial plan just above the periosteum. The key to the mobility of the flap is represented by the release of the corrugator muscle. The flap is rotated and then inset in the defect. The PMFF can be associated with cartilage grafting for composite reconstructions. In some variations of the technique, the pedicle is separated in a second stage debulking the flap excess [18] (Figure 5).

6. *Frontal hairline island flap*: the frontal hairline island flap (FHiF) is a transpositional tunneled island flap harvested from the forehead and nourished by the supraorbital and supratrochlear arteries. It is used for medial canthal, upper nasal dorsum, and nasolabial small–medium defects. The FHiH is harvested in an elliptical shape at the level of the frontal hairline. Once the upper incision is made, the pedicle is dissected subperiosteally toward the orbital rim. The flap is transposed subcutaneously in a tunneled passage to the gap and sutured to the adjacent skin. The donor site is closed by direct suture. This flap represents a tunneled version of the paramedian flap but with indications for smaller skin defects [19] (Figure 6).

7. *Keystone flap*: the keystone area flap (KSAF) is a random-pattern multi-perforator advancement flap applicable to all defect dimensions, as it can be performed in all facial subunits. After tumor excision, the flap is islanded to the subcutaneous tissue with the shape of a Roman arch or vault keystone. The ratio between the width of the defect and the flap should be 1:1. The flap should not be undermined to not injure the multiple perforator vessels. It is important to preserve the superficial venous system and to keep intact the deep fascia. It is divided into four categories: type I is the classic keystone flap, type II includes skin grafting, type III is a double classic keystone, and type IV is based on undermining the skin around the keystone flap. The flap is then inserted into the gap and the closure starts with the edge closer to the defect. The longitudinal angles are closed in a V–Y fashion [8,20] (Figure 7).

8. *Karapandzic flap*: the Karapandzic flap (KF) is a rotation and advancement flap used in the upper lip and commissure reconstructions of full-thickness defects. It can fill medium and large defects. It is based on the facial artery branches. The KF is often harvested bilaterally with two “L-shaped” opposing flaps, eventually of different lengths. Once the upper lip skin defect has been created, a bull-horn skin excision is made in both alar sulci to increase the advancement of the flaps. The flap dissection is performed gently through the skin and subcutaneous tissue to preserve the vascular pedicle and the neural bundle. The two advancement flaps are moved until they meet centrally. Sutures are made by direct closure [21,22] (Figure 8).

9. *Abbè flap*: the Abbè flap (AF) is a rotational flap usually harvested for the reconstruction of the upper lip. Its indications are defects involving 50% or more of the philtrum and defects of the medial element of the lateral subunit greater than 1.5 cm. The AF is considered a cross-lip switch flap, where the donor site is represented by a quadrangular full-thickness portion of the lower lip, frequently in its center–lateral part. The flap is based on the labial artery, the pedicle of the region, which can be medial or lateral in the flap harvested. The AF amount is calculated based on the defect to fill and then transposed with markers on the lower lip. Once the defect in the upper lip is created, the flap is rotated to fill the gap, while the lower lip is closed primarily. This technique temporarily reduces lip movement and mouth-opening due to the closure. The flap requires a second stage of division where the pedicle is cut and the flap is inserted. The flap can also be harvested for defects in the lower lip [23,24] (Figure 9).

10. *Mustardè flap*: the Mustardè flap is a rotational flap adopted for the reconstruction of large cheek defects, the lateral nose wall, and the lower eyelid region. Its harvesting begins from the edge of the excision defect and extends through the lower eyelid to the lateral canthus and ends with an incision superior and lateral to the temple region. The dissection is performed subcutaneously to not injure any facial nerve branches. As the MF is a flap of great dimensions, anchoring it to the zygomatic arch helps to avoid excessive tension on the flap’s insertion. The donor site is closed by direct suture, with the help of Burow’s triangles [25,26] (Figure 10).

## 4. Discussion

**Figure 11:**
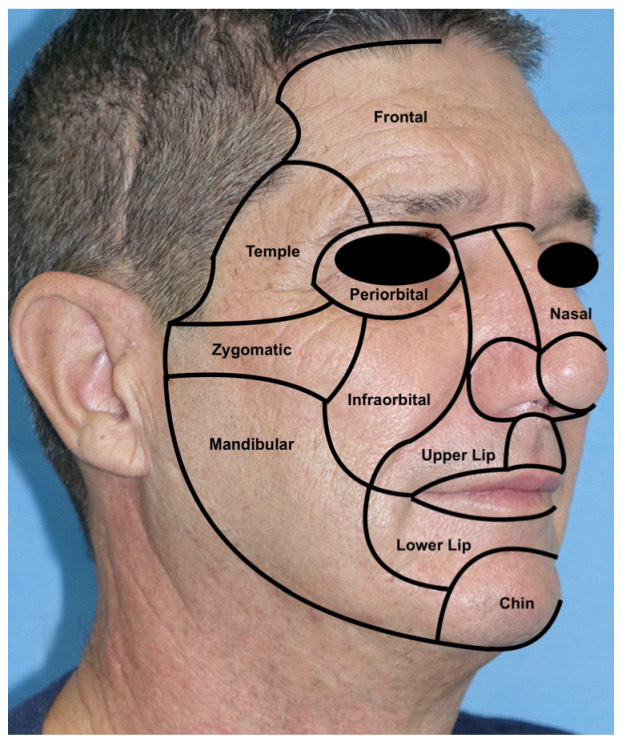
Gonzales-Ulloa aesthetic subunits of the face. From top to bottom: frontal, temple, periorbital, nasal, zygomatic, infraorbital, upper lip, lower lip, mandibular, and chin [4].

The reconstruction of facial defects after skin cancer excision can be challenging, especially in the midface, a region that requires both aesthetic and functional outcomes [23]. The division of the face into facial aesthetic subunits represents a valid method to delimitate the face into regions with their own characteristics and was first introduced by Gonzales-Ulloa in 1956 (Figure 11).

The utility of these subunits is double: on one hand, they delimitate areas with circumscribed histology, thickness, and texture; on the other hand, their separation lines represent marks for surgical incisions to keep areas with different characteristics separated [27]. When defect size and location permit, reconstruction should be carried out within the same subunit or using the most similar one to obtain an excellent texture and functional match [13]. The concept of subunits is the basis of all facial reconstructions, where the restoration must guarantee high results [28].

The gradual but significant evolution of reconstructive techniques has led to several solutions in the hand of a maxillofacial surgeon’s repertoire [3]. The most adopted options are represented by skin grafts, locoregional flaps, and free flaps. Despite skin grafts being versatile, their aesthetic and functional outcomes are very poor. Free flaps are used for very large defects, require the expertise of microvascular surgery, and have longer operative times, which are not suitable for all patients. Pedunculated flaps, also called local or locoregional flaps, are more accessible to surgeons and considered more reliable in specific settings [6]. For these reasons, local flaps are considered the best reconstructive option for small to medium defects [7]. Local flaps are classified on the basis of the method of movement in advancement flaps, pivotal or rotational flaps, transposition flaps, and islanded flaps. Based on their blood supply, they can be divided into random, axial, and perforator flaps [29]. Depending on the distance between the flap and the gap to fill, the pedicle can be identified and moved within the subcutaneous tissue or skeletonized: the pedicle skeletonization increases vascular mobility and adds further degrees of rotation [3,16,17]. In the midfacial region, the main pedicle used is the facial artery and its branches, such as the angular artery, the labial arteries, and the dorsal nasal artery [16,30]. In particular defects, for example in the nasal region, flaps can derive from the forehead vascular plexus, where supratrochlear and supraorbital arteries are the main flap pedicles [18,31]. Doppler probe mapping of vascular pedicles is mandatory during midfacial reconstruction flap design as it allows for the individuation of precise vessel courses [14].

As shown in Table 1, the majority of midfacial flaps are dedicated to cheek and nose reconstruction, as they represent the most prominent areas of the face and, for this reason, are more exposed to traumatic injuries and ultraviolet damage [13,21]. The zygomatic–cheek region is burdened with the need to manage facial nerve branches, protected beneath the SMAS in most locations. A dissection in a plane superficial to that of the SMAS and the parotid–masseteric fascia represents a pillar of fundamental importance in the preparation of flaps in this region [16,29]. While the cheek area is flatter and composed of more similar subunits, the nasal region represents a very complex district. Nasal skin can be tricky during reconstruction: it is thick on the dorsum, tip, and alar region, but becomes thin in the upper dorsum, lateral walls, and columella [32]. Furthermore, the presence of geometrically complex subcutaneous structures such as nasal cartilage is considered a factor that increases the reconstructive difficulty [17]. In 2015, Chang et al. proposed an algorithm for the flap choice in midfacial defect reconstruction to simplify surgical planning. The algorithm was based on two variables: defects size bigger or smaller than 3 cm and their distance from the nasolabial fold. Defects larger than 3 cm require a perforator transposition flap without considering the distance from the nasolabial fold. Smaller defects distant from or just on the fold should be reconstructed with a V–Y advancement flap. Defects smaller than 3 cm and near the nasolabial fold should require a perforator-based transposition flap [15]. Comparing the algorithm with the results of a review of the literature conducted in this study, it is possible to assume that the trend is unchanged: the bigger the flap, the higher the need for a defined vascularization of the flap to guarantee optimal engraftment [33,34].

Locoregional flaps, while safe and reliable, are not without complications. The rate of complications is directly related to flap complexity and the dimension of the defect. Smaller and easier locoregional flaps are the most successful ones. The most common complications are the general ones such as edema, perioperative hemorrhage, hematomas, and infections. Due to the nature of pedunculated flaps, total flap loss is very rare. Partial flap necrosis can occur more often in rotational flaps for pedicle suffering: debridement resolves the issue and allows second-intention healing [9]. Technique complications are represented by pincushioning and standing cone deformities, more common in bilobed and rhombic flaps [10]. Specific regional complications are mostly associated with tension on flaps surrounding tissues, functional deformities, and donor site issues [8]. For example, the cervicofacial and Mustardè flaps, harvested close to the eyelid, are encumbered by the risk of ectropion and eye sagging [10,21]. Paramedian and forehead hairline island flaps are harvested for medium–large defects and their donor site wounds could require a split-thickness skin graft of a dermal substitute to be appropriately closed [18,35,36]. Nasal obstruction is a complication of nasal reconstruction and it is secondary to wound contraction, compromising internal and external nasal valves [31]. Labial flaps such as the Abbe and the Karapandzic flaps can cause functional impairments such as microstomia and lip incompetence to liquids, solvable with minor ancillary surgeries [19,20].

The authors described the most established and standardized techniques for the reconstruction of a complex area. Taking into account the potentially infinite imagination in the design of locoregional flaps, including all of them was conceptually unachievable. For these reasons, the technique selection considered mainstream flaps, not contemplating all possible technical variants and eventually derived techniques. Another limitation of the study concerns the adoption of only one international database as a technique source.

## 5. Conclusions

The midface is one of the most challenging regions to reconstruct for maxillofacial surgeons because of its dynamic functional and aesthetic components. The midfacial district has a complex structure that requires a detailed understanding of the facial subunits, the location and size of the defect, and the choice of the appropriate flap to achieve optimal outcomes. The reconstruction of the midfacial district with locoregional flaps involves a variety of surgical techniques. The use of these techniques requires a great emphasis on pre-surgical planning. In the conducted study, it was found that locoregional pedunculated flaps, due to their great variety, often represent the best solution for midfacial skin defects.

The success of the reconstruction of the midface using locoregional flaps depends on several key elements. One of the most critical elements is pre-surgical planning, which includes a detailed study of the facial subunits to identify the location, size, and extent of the defect. The surgeon must also consider the choice of the appropriate flap, which is crucial in achieving optimal outcomes. This should be carried out while paying attention to the vascular pedicles, which can make a significant difference in the success of the procedure.

In summary, the reconstruction of the midface using locoregional flaps is a complex procedure that requires a great emphasis on pre-surgical planning. Choosing the right flap, respecting the vascular pedicles, and understanding the facial subunits are key elements that determine the success of the procedure. Locoregional pedunculated flaps offer a great variety of options, making them often the best solution for midfacial skin defects.

## Figures and Tables

**Table 1:** Main characteristics of each technique in alphabetical order, divided by type of flap, main midface region, secondary regions, and skin defect size.

Technique Adopted	Type of Flap	Main Midface Region	Secondary Regions	Skin Defect Size
Abbè Flap	Rotational Flap	Upper Lip	-	Small–Medium
Bilobed Flap	Transpositional Flap	Nose	Eyelids	Small
Cervicofacial Flap	Rotational/Advancement Flap	Cheek	Periorbital Region, Preauricular, Neck	Large
Frontal Hairline Island Flap	Transpositional Flap	Nose	Canthal, Upper lip	Medium
Island Composite Nasal Flap	Transpositional Flap	Nose (Higher Portion)	-	Medium
Karapandzic Flap	Rotational Flap	Upper lip	Lower Lip	Medium–Large
Keystone Flap	Advancement Flap	Cheek	Nose, Upper Lip	Medium
Mustardè Flap	Rotational Flap	Cheek	Lateral Nose Wall, Lower Eyelid	Medium–Large
Nasolabial Flap	Advancement Flap	Nose	Periorbital Region, Cheek, Upper Lip	Medium
Paramedian Flap	Rotational Flap	Nose	-	Medium–Large
Retroangular Flap	Transpositional Flap	Cheek	Nose, Periorbital Region, Glabella	Medium
Rhomboidal Flap	Transpositional Flap	Cheek	Scalp, Neck, Nose, Eyelids	Medium

## Data Availability

The data presented in this study are openly available in PubMed.
